# Report of a Case of Inguinal Aneurism

**Published:** 1816-05

**Authors:** J. B. Whitridge

**Affiliations:** the United States Army.


*For the London Medical and Physical Journal.*

*Report of a Case of Inguinal Aneurism;* by J. B. WHITRIDGE, M. D. of the United States Army.

[From the New England Journal of Medicine, &c.]

WILLIAM TROBRIDGE, aged nineteen years, born in the town of Norwalk in Connecticut, of a robust constitution, and by profession a shoemaker, enlisted into the third regiment of artillery at New-York, in August, 1812, from which time he served as a soldier (principally on the frontier), and generally enjoyed good health, until the 7th August, 1814, in attempting to pass the laboratory guard at Sacket's Harbour, (at nine o'clock in the evening,) he was shot by a sentinel on post. From the best information I could obtain of the attending surgeon, and from dissection, a buck shot passed through the dorsum penis, entered the right groin, penetrated the inguinal glands, passed nearly in the direction of the femoral artery, over the os femoris, and lodged in the vastus externus muscle. At the time the wound was received, he is represented to have bled profusely, even to deliquium animi; this, however, soon subsided: he was taken to the Hospital, and superficially dressed,—no hæmorrhagy followed. The wound was treated in the usual manner for gun-shot wounds, and after a lapse of about two months, on the 16th of October, he was discharged from the Hospital, and reported for duty. He performed the ordinary duties of a soldier, until about the middle of December, when he states that he was taken suddenly on post with a pain in his thigh: he reported his situation to the officer of the guard, and was relieved by another sentinel. His thigh was very painful, and, on examination, he found it much swollen. He suffered the pain and swelling to continue a day or two, and then made application to me. He represented it as a recent difficulty, but observed (to use his own expression) that he thought his thigh was going to break out again.

On examination, I noticed his whole thigh to be much tumefied, from his groin almost to his knee, but most about the groin, and particularly at the part where the shot entered. I pressed it on every part with my hand, but could feel no pulsation. The patient being interrogated on this point, observed, that he had felt none at any time, though there was some heat about the tumour, the temperature of which was somewhat above the rest of the body; as there was no redness at the surface, I thought the case a very singular one. The tumefaction being represented as a *recent difficulty*,

*difficulty*, I thought it not improbable, as the pulsation was wanting, which is ever considered an inseparable diagnostic, consequently an infallible criterion of aneurism.* I supposed it the incipient stage of a deep-seated abscess, consequent to the wound as its remote cause, perhaps resulting from a wound of the os femoris by the passage of the buck-shot subjacent to the extensor muscles (that this was not the case was a circumstance at that time unknown to me).

As the tumour seemed rather of an indolent nature, I thought it best to attempt, if possible, to discuss it; and to this end directed an epispastick, of meloes vesicatorii, to be applied; a cathartic of sulph. sodæ to be given him; and ordered him to return to his quarters.

From the pressure of business, I was unable to pay that minute and particular attention which his case merited. I did not see him again until the second day following, at which time he came to me and reported his situation unrelieved—his disease unabated. The blister, it appeared, had done no good, but, on the contrary, had probably done injury.

Considering the case an important one, as he could not receive the necessary attention, nor have the necessary accommodation in the barracks, I ordered him to the Hospital on the 22d of December. For the first day or two after his admission into the Hospital I prescribed nothing for him, but waited to see what course his disease would take. The swelling increased, and the integuments became very tense. I supposed suppuration would take place, and, with a view to promote this end, ordered emollient cataplasms to be applied; these were continued five or six days without intermission.

The effect was, to relax the integuments, to remove the tension of the surface, and to abate the swelling. A fluctuation became in some measure perceptible, though so imperfect as to leave the mind in doubt as to the nature of the tumour, in as much as it did not point at any place, in the usual manner of abscesses, which contain pus. The integuments, however, were thinner at some places than others, and most so at the point where the shot entered; which led to the opinion, that if the abscess contained pus, and were left to itself, it would burst *at that point.* The cataplasms were discontinued after five or six days' application, in consequence of their becoming painful, and disagreeable to the

* Except indeed a case of axillary aneurism described by Mr. Pelletan, and perhaps some others.

patient, having done all that was expected; for it seems that poultices to suppurating wounds or abscesses, or those disposed to suppurate, do no good, after a certain period. A longer continuation of them does material injury. This period must be determined in every case, by the experience and judgment of the practitioner. Although the poultices were discontinued, no pointing had taken place. I applied a flannel roller about the part, and waited five or six days for this event, with an intent to open into the morbid cavity, as soon as the integuments should become thin, and the abscess shew a disposition to evacuate its contents. While deliberating on this, instead of exhibiting any disposition to open, the whole thigh again became swelled and painful; some heat ensued, the parts became rigid, and the fluctuation less perceptible.

The patient constantly lay upon his back, and kept his leg in a state of moderate flexion upon the pelvis, and also at the knee. After the cataplasms were laid aside, I again applied the flannel roller, in order to keep the limb warm; and to prevent the protrusion of the matter upward under Poupart's ligament, and backward among the glutei muscles, I applied it firmly in the groin above the tumour. The health of my patient during this time continued tolerably good; his appetite, however, was poor, and his countenance pallid, in consequence of the pain of his thigh, and of being confined to his bed.

Several days elapsed before any thing was done; the temporary good effects of the cataplasms did not long continue; his thigh again began to swell, and very soon attained to an enormous size. During all this time, no pulsation was perceivable, neither could the tumour be made to recede upon pressure. Although an incision had been proposed, I felt delicate about attempting it, until I had called a consultation, a thing, I am sorry to say, rather unusual in the army, for want of talents and education, and that harmony between my medical brethren, and that confidence in each other, which is so necessary for the advancement of medical science.

Owing to some untoward circumstances, the consultation did not take place until the 8th of January, at which time the integuments had become very thin, much distended, very painful, and almost ready to burst. I then took with me Dr. Thomas Harris, surgeon of the United States navy, an amiable fine young man, a scientific physician, and a well-read surgeon. He had previously heard the history of the *case*, and, on examination, expressed, and appeared to feel much doubt, as to the nature of it; but concurred with me in the opinion, that the tumour most probably contained pus, and and recommended an incision into it, at the thinnest part of its parietes, which was at the point where the shot entered, directly over the crural artery.

We were so far persuaded that pus would be found, on laying open the cavity, that we were unprovided with other instruments than those necessary for this operation. I made an incision into the tumour, through its parietes, three inches and a half in length, and about four-fifths of an inch in depth. Coagulated blood and pus were immediately evacuated, but not freely. I extended the incision upwards of two inches farther; it then not freely evacuating, I commenced clearing it with my thumb and fingers, aided by a pair of common long forceps.

Large portions of very firm coagula were removed, covered with a small quantity of pus on that part which lay contiguous to the parietes of the tumour.

After having removed at least one quart of its black and fœtid contents, a sudden and tremendous gush of fresh arterial blood announced the nature of the disease. I immediately seized the limb, pressed two edges of the incision firmly together, and held them in that situation until I could command the flow of blood by other means. No tourniquet being at hand, I called for a handkerchief, which my assistant, Dr. Harris, applied firmly in the groin, upon the upper part of the tumour, and twisted it up with a stick, which was held in that situation, whilst I laid a large compress of patent lint upon the opening which had been made, and applied a roller about the thigh. The hæmorrhagy being now completely restrained, I had an opportunity for a few moments' deliberation.

The disease was evidently an aneurism of the diffused kind; but whether it was a false aneurism, agreeable to Scarpa's doctrine of all aneurisms, and according to Mr. [now Sir Everard] Home and Dr. Hunter in some cases, or a true aneurism, agreeable to the definition of most writers on this subject, was uncertain, and altogether immaterial, as the treatment is the same.

The sudden loss of blood occasioned a partial syncope, a very great depression of the pulse, and prostration of strength, together with a pale and cadaverous countenance, with rolling of the eyes.

These symptoms were accompanied by a very considerable degree of mental derangement, occasioned by the sudden abstraction of stimuli, which seemed to be a delirium from inanition.

His pulse became almost imperceptible, he fell into a cold sweat, and, in short, his general symptoms were very alarming, ing, and even threatened immediate dissolution, although the quantity of florid arterial blood which he had lost perhaps did not exceed half a pint. Stimulants, such as wine, brandy and water, &c. were given him, which had the effect to raise his pulse a little, and in some measure to restore him, although it did not relieve the delirium. It became necessary immediately to resort to an operation, as the only alternative to preserve the life of the patient, which could be attempted with very little hope of success.

After considering the case, it was finally agreed, as a dernier resort, to take up the external iliac artery as high as possible above Poupart's ligament. Accordingly the necessary preparations were made. I was unprovided with an aneurismal needle of any kind; and, for want of the needle invented by Dr. Physick for securing bleeding arteries in deep narrow wounds, I took a common surgeon's needle, of the largest size, broke off the point of it, reduced its temper by heat, made the end of it smooth, gave it such a curvature as I wished, and adapted it to a pair of common straight forceps, which I substituted for Physick's curved forceps, and tied the handles.

Every thing being now ready, the patient, having previously taken a pill of camphor and opium, was placed upon a table of convenient height, and the operation performed on Sunday, January 8th, at five o'clock in the evening, by candle light, in presence of Dr. Harris and Dr. Gordon of the navy, and a number of gentlemen of the army.

Without entering into a minute detail of the manner of operating, suffice it to say, that the operation was performed in a manner similar to that described by Dr. Dorsey, which was the first and only case in America, that I have seen reported, in which this operation has been performed. The principal difference of this operation was in the situation and extent of the incision. The situation of the artery, of the spermatic cord, and of Poupart's ligament, being previously marked out, I commenced my external incision at a point, upon a line, from the anterior and superior spinous process of the os ilium to the umbilicus, about two inches from the former, and four from the latter. This incision was extended obliquely downward five inches, dividing the skin and adipose membrane, and terminated within one inch of the symphysis pubis. By the subsequent incisions, the tendon of the external oblique muscle, the internal oblique, and the transversalis abdominis were divided; but the incisions were not extended so low as to wound the epigastric artery, or the spermatic cord. I met with considerable embarrassment in this operation, from the delirious state of my patient, who kept kept constantly hallooing, making strong inspirations and expirations, writhing and contorting his body, throwing the abdominal muscles into violent action. But the greatest difficulty I met with was what Mr. Freer so much complains of, that of passing the needle round the artery. This I was unable to do, from the great quantity of adipose substance with which the artery was enveloped, until I had denuded the artery by careful dissection, and a division of the fascia. This part of the operation was the more dangerous and difficult, in as much as I was under the necessity of performing it by candle-light.

The strength of the patient was preserved during the operation by the repeated administration of stimulants; and, after it was completed, the handkerchief and the bandage were removed from the thigh: that part of the coagula which protruded immediately at the orifice of the sack was also removed, the limb washed, and superficially dressed, by the application of a large pledget of lint, and a roller. The patient was then placed in bed, with his leg in a relaxed position, supported by pillows.

The patient, though somewhat restless, passed the first night as comfortably as could be expected under existing circumstances. The relative heat of the lower extremities was not observed previous to the operation. The thermometrical temperature after the operation, at twelve o'clock in the evening, when I made the experiment, was as follows:

| | |
|---|---|
| Temperature of the room | 66° |
| ---------- under the tongue | $97\frac{1}{3}$ |
| ---------- of the right ankle, or the ankle of the aneurismal limb | 86 |
| ---------- of the left ankle | $88\frac{1}{2}$ |

*Monday, January 9th.*—At five o'clock and twenty minutes, A.M. examined the temperature between the toes, and repeated the experiment at the ankles.

| | |
|---|---|
| Temperature between the toes of the right foot | 85° |
| ---------- left foot | 94 |
| ---------- of the right ankle | $90\frac{1}{3}$ |
| ---------- of the left ankle | 90 |

Temperature of the room being about the same as before.

At eight o'clock my patient was perfectly calm and quiet, but recollected nothing that had passed since the laying open of his thigh. The blood was constantly oozing from the orifice of the tumour, which kept the dressings and cloths which were laid under it quite wet. At twelve o'clock I took off the dressings from the thigh (but left those about the body undisturbed), and removed that portion of coagula which which lay contiguous to the incision. At twenty minutes past three o'clock, P.M.

Temperature of the room ---------------------- 66°
----------- under the tongue -----------------100
----------- aneurismal limb at the ankle ------ 92
----------- left leg at the ankle --------------- 93

No considerable variation in the evening.

He remained very quiet through the day, but felt no disposition to sleep at evening. At ten o'clock I gave him half a grain of opium, which had but little effect; at two o'clock, a pill containing one grain and a half of camph. and half a grain of opium was given him, which procured some sleep: as he had had no evacuations from the bowels since the operation, to prevent the constipating effect of the opium, at four o'clock, I directed half an ounce of ol. ricini to be given him.

*Tuesday, January 10th.*—Some difficulty of respiration, pulse full and hard, heat somewhat increased; ordered sub. muriatis hydr. gr. v. rhei. gr. x. which produced several dejections in the course of the day, without much alleviation of the symptoms. In the evening there was quite an increase of heat, which was as follows:

At 11 o'clock in the evening, temperature of the room ------------------------------------------ 67°
Temperature under the tongue -----------------100
----------- right leg above the knee ----------- 96
----------- left ditto ditto --------------------- 97
----------- between the toes of right foot------ 96½
-------------------------------- left foot ------ 98

The thigh was dressed as yesterday, and at least one pint of coagulated blood removed, by my thumb and fingers, and by pressure upon the thigh. At twelve o'clock at night, bled him oz. 14, which gave him some relief, but occasioned no sleep: at two, he took a pill of one grain and a half of camphor and half a grain of opium, after which he dosed until morning.

*Wednesday, January 11th.* Morning.—Patient somewhat better; complains of much hoarseness, and a dry throat; directed syr. scill. marat.

At half-past two o'clock again examined the heat of the body.

Temperature of the room being---------------- 63°
----------- right leg above the knee ----------- 97
----------- left ditto ditto --------------------- 98
----------- between the toes of the right foot -- 94
---------------------------------- left foot ---- 95

Arterial action something above the healthy standard, complains plains of some tightness across the thorax; having no alvine dejection, at eleven o'clock in the evening, gave him gr. iii. calomel and gr. iv. of rheum. He has complained of considerable thirst for two or three days: his principal drink has been lemonade; food—water-gruel, milk-porridge, crust coffee, &c.

*Thursday, January 12th.*—The cathartic of the preceding evening not operating, at twelve o'clock, ordered half an ounce of ol. ricini, which produced four or five evacuations, and a considerable reduction of the pulse. The temperature of the extremities was also reduced two degrees lower than yesterday.

*Friday, January 13th.*—Condition of the patient much the same as yesterday; flattering prospect of recovery. At 10 o'clock in the evening, after dressing—

Temperature above each knee ---------------- 94°
---------- between the toes of the right foot -- 94°
------------------ ----------- left foot ---- 96

At eleven o'clock directed tincturæ opii. gutt. xv.

*Saturday, January 14th.*—The anodyne, not having the desired effect, at 10 o'clock in the morning, ordered a repetition of the dose, and, at six, an aperient of ol. ricini; syr. scillæ mara. continued. An anodyne in the evening. The aneurismal sack, which has now become a complete abscess, has been dressed daily since the operation; and the incision at the abdomen has also been dressed daily since the third day after the operation. The dressings are strips of adhesive plaster, a pledget of lint, and the uniting bandage. At the first dressing, the angles, and a considerable extent of the incision, had united by the first intention.

*Sunday, January 15th.*—On dressing the abscess to-day, pressed out a large quantity of coagulated blood, mixed with pus, which seemed to relieve a little hacking cough which has affected him for two or three days past. Pulse above ninety in a minute, much reduced in force, yesterday and to-day, but not much in frequency. For want of a time-piece denoting seconds, I was unable to ascertain the frequency of the pulse with that particular accuracy which might have been wished. At ten o'clock in the evening he took twelve drops of laudanum, after which he slept better than any preceding night since the operation.

*Monday, January 16th.*—On removing a strip of adhesive plaster, the aneurismal sack burst upon the top of the thigh, at the place where the shot entered. Grumous blood and pus spouted at least four inches from the orifice, which, at first, excited some surprise, as it had a little the appearance of arterial blood. At least one pint of coagula was removed at this dressing, by my thumb and fingers, together with my knife and scissors, principally at the external orifice. Heat and pulse much reduced at this dressing. Ordered one table-spoonful of the vinous tinct. of bark. Bowels somewhat constipated, nothing having passed for the last thirty hours. Ordered an enema of sulph. sodæ to be administered; pulse near one hundred in a minute.

*Tuesday, January* 17*th*.—Abscess dressed twice; pulse very much reduced; patient much affected with cough. Ordered bark and wine to be given every hour, and an anodyne in the evening.

*Wednesday, January* 18*th*.—Again repeated the thermometrical experiment:—

| | |
|---|---|
| Temperature of the room at one o'clock | 68° |
| ——— under the tongue | 100 |
| ——— between the toes of the right foot | 93 |
| ——— left ditto | 94½ |
| ——— above the right knee | 97 |
| ——— left ditto | 98 |

Bark and wine continued hourly, through the day. Cough increases, with a hectic flush in the afternoon. Bowels constipated. Ordered an enema of amylum in the evening. Pulse quick and fluttering; an hundred in a minute. At ten o'clock gave an anodyne as usual.

*Thursday, January* 19*th*.—Appearances much the same as yesterday. Had a voluntary stool in the evening; medicines continued; extremities cold; right foot painful; a want of action in the toes; directed the foot to be bathed with brandy and the tinc. opii.

*Friday, January* 20*th*.—Cough somewhat abated; rested well the preceding night; tongue less coated; pulse good, about eighty in a minute. Complains of severe pain in the knee, calf of the leg, and foot, both at and after dressing. Heat much diminished; the right foot indicating a disposition to mortification:—

| | |
|---|---|
| Temperature under the tongue | 99½° |
| ——— above the right knee | 94° |
| ——— left ditto | 95 |
| ——— between the toes of the right foot | 65 |
| ——— left ditto | 84 |

Medicines continued; hot bricks applied to the feet; bathing and friction also continued.

*Saturday, January* 21*st*.—Appearances of the wound occasioned by the operation, and the abscess, good; discharges healthy pus from both, but much more from the latter than at any time previous. The foot and leg very painful, and swelled, with a livid appearance, mortification having commenced menced in the little toe. Temperature of the foot much below the rest of the body. Ordered ten drops of laudanum to be given three times a-day, and the bark and wine continued in increased quantities. The aneurismal limb bathed as before, and bandaged with a flannel roller. At ten o'clock in the evening, bathed the right foot and ankle with a saturated tincture of cantharides, which produced an efflorescence of the skin, but did not materially increase the heat.

*Sunday, January 22d.*—Coldness of the foot and ankles continues. Mortification progresses in the toes. Partial sweats, particularly about the face, hands, and neck. Pulse strong and good, eighty in a minute. From the coldness of the weather, and the openness of the Hospital, it was very difficult to keep the room of an equable temperature:—

| | |
|---|---|
| Temperature of the room | 49° |
| ———— at the right ankle | 63 |
| ———— left ditto | 84 |
| ———— between the toes of the right foot | 56 |

At eight o'clock in the morning, ordered the right foot and ankle to be bathed with ol. teribinth, after which to be bathed hourly with a saturated tincture of cantharides.

*Monday, January 23d.*—From the internal exhibition of medicine, and from the local applications that were made, the heat of my patient is very much increased:—

| | |
|---|---|
| Temperature of the room at one o'clock | 49° |
| ———— under the tongue | 103 |
| ———— between the toes of the right foot | 64 |
| ———— at the right ankle | 64 |

The variation of heat is dependant upon a variety of circumstances, particularly that of dressing the patient; the exposure which it occasions uniformly produces a reduction of temperature. Pulse good; medicines continued. Though the action of the foot is somewhat increased, not being blistered by the tincture of cantharides, I applied a vesicating plaster over the whole foot and ankle. From fear that the pressure of the dressings upon the thigh might contribute to produce the coldness and swelling of the foot, the bandages were loosened last evening. To-day the suppuration is very extensive, probably in consequence of loosening the dressings; nearly half a pint of bland healthy pus discharged. The os femoris is discovered to be bare for two or three inches, and the periosteum to be destroyed. Firm and tight dressings were again applied. The ligature upon the artery came away this day, and the wound, occasioned by the operation, appears well, having completely healed, except the little place occasioned by the ligature.

*January 24th, 25th, and 26th.*—Colliquative sweats supervene.

vene. The situation of the patient now becomes quite precarious. Tonics and stimulants are continued in large quantities, with the addition of columb. and acid. sul. aromat.; injections are administered according to circumstances. The blistering is continued upon the foot and leg, without arresting the progress of the mortification. By these means the action of the system is kept up. Considering the situation of the patient, his pulse continues remarkably good. Appetite, which has hitherto been very poor, is now a little better. Food principally milk-porridge, occasionally a little chicken-tea or beef-soup.

*Friday, January* 27*th.*—The wound at the abdomen is now quite well. Cough increases; pulse feeble, frequent, and quick. A copious discharge of pus from the abscess, at least half a pint, which does not appear so well. It is thin, flaky, and smells disagreeably. The heat of the aneurismal limb, as far as the knee, somewhat increased, and about the same as the other. At ten o'clock in the evening—

| | | |
|---|---|---|
| Temperature of the right knee | ---------------- | 96° |
| ———————— left ditto | ------------------ | 97 |

Vesicating plasters and other medicines continued.

*Saturday, January* 28*th.*—Pulse stronger, and much less frequent. Appetite somewhat increased. Expectoration considerable. Had two voluntary discharges from the bowels. The discharge from the abscess not so great; appearance of the pus much better, quite homogeneous, and perfectly bland. The blisters, having now drawn tolerably well, are removed, and the parts dressed with basilicon sprinkled with cantharides.

*January* 29*th and* 30*th.*—Appearances much the same. Bowels, which have all along been constipated, are now becoming rather loose. Appetite better; ate some beef-steaks to-day (Monday) with great relish. The gangrenous process still progressing. Medicines continued half-hourly, in as large quantities as the stomach will bear, and as much wine and brandy as the patient will take.

There has, apparently, been a great want of action in the parietes of the aneurismal sack and parts adjacent, for a long time; perhaps owing to the pressure of firm coagula, so as to destroy the action, and produce a sloughing of the neighbouring parts. With the coagula, some portions of mortified substance were discharged daily, which, from their near resemblance, it was difficult to distinguish. To obviate this, after syringing the abscess with soap and water, and washing it with diluted brandy, I dressed it several times with cinch. off. in its pulverulent state, which, I think, acts both as a tonic and absorbent in abscesses and ulcers. How far far it was beneficial in this case, is difficult to determine; in other cases, I have found the external application of it particularly serviceable.

*Tuesday*, 31*st.*—The hole, at the point where the shot entered, has closed. The discharge of pus continues copious, say half a pint per diem. The foot and leg worse, especially the latter, which has become livid, quite to the knee, with a fluctuation about the capsular ligament. Medicines continued.

*Wednesday*, *February* 1*st.*—Appearances of the patient much worse. Sphacelation progresses rapidly. Leg very much swollen, and discharges a bloody watery fluid. The temperature of the foot and leg reduced to an equilibrium with the surrounding atmosphere. At one o'clock P.M. applied a blister above the knee, and a fermenting cataplasm to the leg and foot, with the hope of arresting the progress of mortification; but all to no purpose. The abscess this evening discharges a thin, bloody, fœtid matter; the external opening much dilated, and the parts within discoloured. Pulse small and frequent, about one hundred and thirty in a minute. Appetite failing; medicines continued as before.

*Thursday*, *February* 2*d.*—Mortification and swelling increase and extend quite to the abdomen. Scrotum discoloured and suffused with bloody lymph. Partial sweats continue. Pulse rapid and almost imperceptible. Countenance much altered, but cheerful. Very little appetite; ate some roasted apples to-day with relish. The yeast poultices are discontinued, and the limb sponged with a tincture of myrrh; afterwards covered with black oxyd of carbone and bandaged. Medicines, brandy, &c. continued, in as large quantities as the stomach will bear.

*Friday*, *February* 3*d.*—Patient still lives; appearances much the same as yesterday; the whole abdomen much swollen. Loaths both food and medicine. Stimulants, however, are continued in large quantities, and brandy in particular every few minutes.

*Saturday*, *February* 4*th.*—At five o'clock in the afternoon, under the treatment of yesterday, my unfortunate patient died, retaining the full exercise of his intellectual powers until the last.

On Sunday morning, in presence of Dr. Harris, I examined the body of the deceased, and am happy to state to the world, although the event of this case was unfortunate, that so far as relates to the operation all was well. It succeeded most perfectly.

*Appearances on Dissection.*—All the parts in the neighbourhood of the artery were uninjured by the operation, except those necessary to be divided. The inflammation in the cavity of the wound produced a slight adhesion of the omentum to the peritoneum, say from one third to half an inch. Except this morbid adhesion (which was not of the least consequence) all the parts were nearly in their natural state. The internal iliac artery was not so much enlarged as might have been expected. From the putrid state of the limb, the texture of which was very much destroyed, I was unable to trace the minute ramifications of the internal iliac artery into the substance of the thigh, but presume, from the size of the trunk, which was very little larger than its fellow, that they were not much dilated. I have now before me a preparation of the artery and vein, with the trunk of the principal branches of the artery, from the bifurcation of the aorta to the popliteal artery of the ham, with the external and internal iliac of both sides.

The disease was evidently a false aneurism, an aneurism of the diffused kind. The coats of the artery were not in the least dilated; the bleeding point of the artery was four and an half inches from the bifurcation of the arteria iliaca communis. The mouths of the divided artery were an inch and an half asunder, gaping, with their edges ragged. The ligature was made one inch below where the internal iliac is given off, which, of course, divided the artery; the mouths of which, at this place, were separated half an inch, standing open, their edges smooth. The accompanying vein and nerve were sound. The thigh very much reduced in size, being diminished full three-fourths since the operation, by the general wasting of the system, the absorption of the parietes of the sack, and the sloughing of the muscles. The periosteum destroyed for a considerable extent, and the os femoris carious.

*Queries.*—What was the cause of this aneurism? Did it result from a direct wound of the artery, by the passage of the buck-shot? by an erosion of the artery subsequent to that period? or by some cause independant of either?

Did the mortification depend upon the obstruction of the circulation, occasioned by the operation? or by the destruction of the minute ramifications of the internal iliac artery, occasioned by the pressure of the aneurismal tumour upon the surrounding parts, and by the subsequent sloughing?

Might not this patient have been saved by an earlier operation?

*Sacket's-Harbour; February* 22, 1815.

